# Neurodegenerative Diseases: An Overview of Environmental Risk Factors

**DOI:** 10.1289/ehp.7567

**Published:** 2005-05-26

**Authors:** Rebecca C. Brown, Alan H. Lockwood, Babasaheb R. Sonawane

**Affiliations:** 1 Association of Schools of Public Health, National Center for Environmental Assessment, Office of Research and Development, U.S. Environmental Protection Agency, Washington, DC, USA; 2 Departments of Neurology and Nuclear Medicine, Veterans Affairs Western New York Healthcare System and University at Buffalo, The State University of New York, Buffalo, New York, USA; 3 National Center for Environmental Assessment, Office of Research and Development, U.S. Environmental Protection Agency, Washington, DC, USA

**Keywords:** Alzheimer disease, amyotrophic lateral sclerosis, electromagnetic fields, metals, multiple system atrophy, neurodegeneration, Parkinson disease, pesticides, progressive supranuclear palsy, solvents

## Abstract

The population of the United States is aging, and an ever-increasing number of Americans are afflicted with neurodegenerative diseases. Because the pathogenesis of many of these diseases remains unknown, we must consider that environmental factors may play a causal role. This review provides an overview of the epidemiologic evidence for environmental etiologies for neurodegenerative diseases such as Alzheimer disease, Parkinson disease, parkinsonian syndromes (multiple system atrophy and progressive supranuclear palsy), and amyotrophic lateral sclerosis. Epidemiologic evidence for an association between environmental agents’ exposure and neurodegenerative diseases is not conclusive. However, there are indications that there may be causal links, and the need for more research is obvious.

The population of the United States is aging, and an ever-increasing number of Americans are afflicted with neurodegenerative diseases. Neurodegenerative diseases result from the gradual and progressive loss of neural cells, leading to nervous system dysfunction. According to the National Institute of Neurological Disorders and Stroke, there are more than 600 neurologic disorders, with approximately 50 million Americans affected each year.

These diseases cost the U.S. economy billions of dollars each year in direct health care costs and lost opportunities; it is estimated that $100 billion per year is spent on Alzheimer disease (AD) alone (Meek et al. 1998). In addition to the financial costs, there is an immense emotional burden on patients and their caregivers. As the number of elderly citizens increases, these costs to society also will increase.

Until recently, most concerns about environmental agents have centered on their potential for causing cancer. Cancer and neurodegeneration represent opposite ends of a spectrum: whereas cancer is an uncontrolled proliferation of cells, neurodegeneration is the result of the death of cells, whether due to direct cell death by necrosis or the delayed process of apoptosis. Attention is now being focused on environmental agents’ potential for damaging the developing and mature nervous system, resulting in neurodegenerative diseases.

Known risk factors for neurodegenerative disease include certain genetic polymorphisms and increasing age. Other possible causes may include gender, poor education, endocrine conditions, oxidative stress, inflammation, stroke, hypertension, diabetes, smoking, head trauma, depression, infection, tumors, vitamin deficiencies, immune and metabolic conditions, and chemical exposure. Because the pathogenesis of many of these diseases remains unknown, we must consider the role of environmental factors in these diseases.

In this review we examine the human evidence for environmental etiologies for some diagnosed neurodegenerative diseases. Epidemiologic literature, not case studies or experimental animal research, was searched for relevance to an association between neurodegenerative diseases and environmental agents. We briefly review genetics and lifestyle habits (e.g., smoking, coffee, alcohol) but not other potential risk factors such as age at onset, socioeconomic status, gender, ethnicity, and education. Although it is acknowledged that the etiology of neurodegenerative diseases is often multifactorial (particularly gene–environment interactions), the purpose of this review is to examine the extent of the available epidemiologic literature solely on environmental agents.

## Study Type Selection

The greatest assurance for causality comes when the exposure to the environmental agent can be determined before the outcome. Because of the nature and variety of neurodegenerative diseases, the vast majority of cases are observed in the elderly population, yet the exposure could have occurred years or decades before the resulting effect. This long latency period makes it extremely difficult to track exposures before the outcome in a longitudinal fashion. A few groups have conducted studies of this type (Abbott et al. 2003; Baldi et al. 2003b; Hernan et al. 2001; Lindsay et al. 2002; Petrovitch et al. 2002; Preux et al. 2000; Rondeau et al. 2000; Tyas et al. 2001).

Because of these major constraints, many investigators have used a case–control study design that examines cases after diagnosis. The major limitation of this type of study is recall bias (see “Bias”). Ecologic studies are another type of study, with the major limitation being the inability to characterize exposure data to individuals (see “Exposure Definition”).

## Disease Definition

Precise determinations of neurodegenerative disease can be elusive, although for approximately 80% of cases, a diagnosis made by a clinician is confirmed postmortem (Mok et al. 2004). For many cases, a confident diagnosis can be made only during an examination of brain tissue after death. Because autopsies are not always conducted, when using mortality data such as death records, there are obvious problems if the neurologic disease in question is not listed as a primary or secondary cause of death (Ritz and Yu 2000). Most studies focus on symptoms ascertained during life, and many of the criteria used to define neurologic disease are left to a certain extent to subjectivity, with inevitable associated misclassification of disease.

Also, there are difficulties associated in accurately differentiating one neurologic disease from another, particularly since many conditions may co-exist. For example Drayer et al. (1986) measured “Parkinson plus syndrome,” which was defined as Parkinson disease (PD) and multiple system atrophy (MSA), or PD and progressive supranuclear palsy (PSP). There is also co-morbidity of vascular conditions such as stroke or coronary heart disease. To complicate matters further, diagnostic criteria have changed or evolved over time. For example, those for MSA were revised as recently as 1998 (Wenning et al. 2004).

One solution to the problems associated with diagnosing neurodegenerative diseases would be to measure neurologic symptoms individually and early, before clinical diagnosis. Formal neuropsychologic tests can provide some objective support. Several lines of evidence have been published with this focus (Baldi et al. 2001; Engel et al. 2001; Farahat et al. 2003; Kamel and Hoppin 2004; Kamel et al. 2003; Pilkington et al. 2001). However, there may be some uncertainty with linking early subtle effects with clinical diagnosis. For this reason, we examine in this review only those studies where there was definitive clinical diagnosis for neurodegenerative disease.

## Exposure Definition

A number of studies have examined the relationship between exposure to environmental agents and neurologic outcomes. Many studies involve a plethora of individual agents as a mixture, so it is understandable that some have not found an association for neurologic outcomes using a broad exposure definition. Similarly, the grouping of pesticides as an exposure category may be entirely too large. Many researchers have attempted to subdivide this class of chemicals into insecticides, herbicides, and fungicides, but even then a large number of substances could be classified within these clusters, and it remains difficult to tease out the associated environmental agent with the adverse outcome. For example, Baldi et al. (2003b) note the lack of information on specific pesticides because of trade secrets, although they could surmise the pesticides most often used in vineyards would be fungicides.

Accurate exposure assessment may be difficult to perform because of the retrospective aspect of case–control studies (see “Bias”). Also, assigning exposure categories may result in misclassification for ecologic studies (Gauthier et al. 2001; Ritz and Yu 2000) and when proxy respondents are used (Gauthier et al. 2001). There is often a lack of dose–response data, and similarly, the intensity of exposure may be missed. In environmental exposure data, there may be a peak exposure not captured in averaged exposure data, or there may be an accumulation of low-level exposure that may be below the detection limit. For biomonitoring data, nonpersistent exposures will be difficult to capture with accuracy, and the organ or tissue assessed may or may not be where the chemical is deposited or where it has an effect. Finally, if for some exposures there is an earlier critical window of exposure that sets the stage for later neurologic degeneration, a focus on later exposure history may not capture early developmentally relevant exposures.

## Power

To find statistically significant results, investigators must have a sufficiently large study sample, or once the data are stratified the results will not be very telling. Likely this is why many investigators publish results on groups of chemicals (i.e., pesticides) rather than on specific chemicals, or on diagnosable diseases rather than on individual symptoms. Therefore, it is not surprising that little evidence exists to support an association between PD and specific pesticides (Engel et al. 2001; Hertzman et al. 1994). One study in particular did find an association for paraquat and PD (Liou et al. 1997), and another found an association between organochlorines and alkylated phosphates and PD (Seidler et al. 1996). However, the number of studies examining specific pesticides resulting in statistically significant conclusions is limited.

Additionally, many of the case–control studies use small numbers of study participants, particularly those studies measuring biomarkers of exposure (Bergomi et al. 2002; Drayer et al. 1986; Felmus et al. 1976; Fleming et al. 1994; Gellein et al. 2003; Kapaki et al. 1989; Kasarskis et al. 1995; Miyata et al. 1983; Moriwaka et al. 1993; O’Mahony et al. 1995; Rajput et al. 1986; Sood et al. 1990; Stober et al. 1983; Vinceti et al. 2002; Yasui et al. 1991a, 1991b, 1993). This limits the amount of power to detect a statistically significant association. Also, although not discussed in this review, the increasing focus on gene–environment interactions will have a major impact on the results because of the need to stratify results across genotype as well as chemical type.

## Bias

Closely related to exposure assessment, a major difficulty for case–control studies generally is recall bias. Because the exposures tend to occur much earlier than the diagnosed outcome, the individual is asked to remember potential exposures over a long period; this is exacerbated for neurological diseases because the outcomes in question also tend to affect memory. However, those individuals with symptoms may overreport exposures because of the desire to determine a cause for their condition.

Selection bias may occur if those participating in the study are healthier than those who do not participate. This is known as the “healthy worker effect” because many occupational studies contain only those employees who have remained healthy and able to retain their job. For nonoccupational studies, those with more severe neurodegenerative diseases may participate to a lesser degree because of their reduced ability to communicate and travel to study centers.

## Confounders

As mentioned above, other risk factors for neurodegenerative disease may include gender, endocrine conditions, oxidative stress, infection and inflammation, nutrition, vascular conditions, depression, head trauma, tumors, and level of education. Ethnicity and culture may also have implications and provide insights into the etiology of some diseases (Marder et al. 1998). In particular, smoking and caffeine and alcohol consumption have been postulated to have an association with neurodegenerative diseases.

Although the protective effect of smoking on PD is well known (Allam et al. 2004; Quik 2004; Ross and Petrovitch 2001), there is conflicting epidemiologic evidence regarding an association between smoking and risk of AD (Almeida et al. 2002; Letenneur et al. 2004). There is much less evidence for an association between smoking and parkinsonian syndromes, but one study showed a protective effect for MSA but not for PSP (Vanacore et al. 2000).

Similarly, caffeine consumption has generally been shown to be protective against the development of PD (Ross and Petrovitch 2001), although there is much less consistent information for an association with AD (Lindsay et al. 2002; Tyas et al. 2001). There is mixed evidence for a protective effect of alcohol and PD (Behari et al. 2001; Benedetti et al. 2000; Checkoway et al. 2002; Fall et al. 1999; Kuopio et al. 1999; Liou et al. 1997; Morano et al. 1994; Paganini-Hill 2001; Wang et al. 1993) and less for alcohol and AD (Letenneur et al. 2004; Tyas 2001).

## Alzheimer Disease

Alzheimer disease is perhaps the prototypical degenerative disease affecting the central nervous system. AD is a chronic progressive disease characterized by memory loss and deficits in one or more of the following cognitive domains: aphasia (language disturbance), agnosia (failure to recognize people or objects in presence of intact sensory function), apraxia (inability to perform motor acts in presence of intact motor system), or executive function (plan, organize, sequence actions, or form abstractions). In addition, these deficits must be severe enough to interfere with daily life or work, and they must represent a significant decline from an earlier level of function. It is estimated that about four million Americans are currently diagnosed with AD. The prevalence rate is about 7% for individuals aged 65 or more, with the risk doubling every 5 years after age 65 (McCullagh et al. 2001; McDowell 2001).

Although most cases of AD are thought to be sporadic, there are at least four well-known risk factors for AD: increasing age, familial association, Down syndrome, and the apolipoprotein E4 allele (Cedazo-Mínguez and Cowburn 2001; McCullagh et al. 2001; Raber et al. 2004; Rubinsztein and Easton 2000; Weisgraber and Mahley 1996). Some examples of the association betwen exposure to environmental agents and AD are described briefly below.

Heavy metals are well-recognized environmental agents that affect brain development, leading to life-long impairment. Several epidemiologic studies have examined the possible link between aluminum (Al) and AD, with conflicting results. One study found Al in antiperspirants to be significantly associated with AD (Graves et al. 1990), but others showed no association for Al in antiperspirants (Lindsay et al. 2002) or in antacids (Broe et al. 1990; Graves et al. 1990; Lindsay et al. 2002; Tyas et al. 2001). Studies examining occupational exposures of Al found slightly elevated but nonsignificant risk (Graves et al. 1998; Salib and Hillier 1996) or no association (Gun et al. 1997). Also, no association was observed for Al in bone and AD (O’Mahony et al. 1995). One reason for this discrepancy in occupational studies is the inaccurate exposure assessment based on job description and rated for exposure to metals (Graves et al. 1998). Furthermore, these comparisons may not be appropriate because of the different types of exposures, for example, dermal, oral, and inhalation.

The findings for an association between Al in drinking water and AD are also inconclusive. Two studies demonstrated statistically significant results (McLachlan et al. 1996; Rondeau et al. 2000), although the latter did not find a dose–response relationship. Others have found no association (Forster et al. 1995; Martyn et al. 1997). Because of this variability, some researchers are now attempting to explore whether speciation of Al plays any role in causation of AD. For example, Gauthier et al. (2000) found an association with monomeric organic Al in drinking water and AD but not with other forms of Al.

Metals other than Al have also been studied for the relationship with AD but to a much lesser extent. No association was observed for occupational exposure to lead or mercury (Gun et al. 1997), mercury from dental amalgams (Saxe et al. 1999), or increased mercury in the pituitary gland of AD cases versus controls (Cornett et al. 1998a). However, a non-significant elevation of mercury in the brain was associated with AD (Cornett et al. 1998b). Similarly, a statisticially significant association was observed between AD and an elevation of iron in the brain (Cornett et al. 1998b) but not in the pituitary gland (Cornett et al. 1998a), although these studies may have suffered from low power. Related to the storage of iron, an increase of the protein ferritin was found to be higher in the cerebral spinal fluid of patients with AD than that of controls (Kuiper et al. 1994). A statisticially significant association was found between AD and an elevation of zinc in the brain (Cornett et al. 1998b) but not for concentrations of zinc in hair and serum (Shore et al. 1984) or pituitary gland (Cornett et al. 1998a), although these studies may have suffered from low power. No association was observed for hair and serum concentration of copper or magnesium and AD (Shore et al. 1984). An association was observed between AD and increased selenium levels in the brain (Cornett et al. 1998b) but not in the pituitary gland (Cornett et al. 1998a).

The neurologic effect of some pesticides (especially organophosphates and carbamates) on their intended targets is known, and the evidence is growing for the unintended consequences on humans. A significant association was observed between occupational exposure to pesticides in general and AD (Baldi et al. 2003b), and statistical significant risk was observed for fumigants and defoliants and AD (Tyas et al. 2001). Others have not found an association for occupational (Baldi et al. 2003b; Gun et al. 1997; Tyas et al. 2001) or residential exposure to pesticides and AD (Gauthier et al. 2001). Fleming et al. (1994) examined the biologic burden of pesticides in AD cases and found an association between pesticides measured in the brain and AD.

Electromagnetic fields (EMFs) have been suspected as a causal factor for the development of AD. Sobel et al. (1996) found a strong association for EMFs and AD, with an increased risk for males compared with females. Feychting et al. (1998) found a strong but non-significant association with AD. A dose–response trend was observed for both outcomes. However, Graves et al. (1999) observed no association between EMF exposure and AD.

The few studies examining solvent exposure and the development of AD are contradictory. One study using job description as proxy for exposure assessment found a non-significant but suggestive association (Graves et al. 1998), yet other studies (Gun et al. 1997; Tyas et al. 2001) and a meta-analysis found no association (Graves et al. 1991).

## Parkinson Disease

Parkinson disease is unique from AD in that it is characterized by abnormalities of motor control, as opposed to intellectual and personality changes. PD is characterized by resting tremors, bradykinesia (slowness of voluntary movement), rigidity, and a loss of postural reflexes. Patients with PD typically have a flat, expressionless face and walk with a stooped gait characterized by small steps. Many patients also experience severe depression.

The lifetime risk for PD is estimated to be 2 and 1.3% for men and women, respectively, and between 3.7 and 4.4% for “parkinsonism,” a term used to characterize other clinical conditions characterized by akinesia and rigidity that do not meet clinical or pathologic criteria for idiopathic PD (Elbaz et al. 2002). Although a number of genetic polymorphisms are linked to PD (Checkoway et al. 1998), it is likely that the majority of cases of PD are not inherited but related to environmental factors. This is supported by a genetic study of twins by Tanner et al. (1999), who observed that monozygotic–dizygotic concordance rates are indistinguishable, implying a lack of genetic influence and a strong probability of an environmental influence. Examples of environmental risk factors for PD are discussed below.

Substantial numbers of epidemiologic studies have found positive associations between PD and exposure to pesticides (Abbott et al. 2003; Baldi et al. 2003a, 2003b; Fall et al. 1999; Herishanu et al. 2001; Hertzman et al. 1994; Liou et al. 1997; Petrovitch et al. 2002; Ritz and Yu 2000; Seidler et al. 1996), herbicides (Butterfield et al. 1993; Gorell et al. 1998; Semchuk et al. 1992, 1993), insecticides (Butterfield et al. 1993; Gorell et al. 1998), and for residence in a fumigated home (Butterfield et al. 1993). Similarly, Fleming et al. (1994) found increased levels of pesticides in the brains of PD cases versus controls.

There are also a number of studies that have shown no association between PD and pesticide exposure (Behari et al. 2001; Koller et al. 1990; Kuopio et al. 1999; McCann et al. 1998; Smargiassi et al. 1998; Stern et al. 1991), herbicide exposure (McCann et al. 1998; Smargiassi et al. 1998), and fungicide exposure (Gorell et al. 1998). Some studies use a broad exposure definition for pesticides, as mentioned above.

Farming occupation and farm residence are related to pesticide exposure. A significant association was found for PD and farmworkers (Gorell et al. 1998; Ho et al. 1989; Wechsler et al. 1991) and orchard workers (Hertzman et al. 1990). However, these positive findings have not been replicated by other investigators examining farming (Baldi et al. 2003b; Behari et al. 2001; Chan et al. 1998; Engel et al. 2001; Koller et al. 1990; Kuopio et al. 1999; Morano et al. 1994; Rocca et al. 1996; Wong et al. 1991) or residence on a farm (Butterfield et al. 1993; Gorell et al. 1998; Semchuk et al. 1991).

Residence in rural locations was found to be associated with PD in a number of studies (Butterfield et al. 1993; Golbe et al. 1990; Ho et al. 1989; Koller et al. 1990; McCann et al. 1998; Morano et al. 1994; Rajput et al. 1986; Stern et al. 1991; Wong et al. 1991). Yet no association was found in a few other studies (Baldi et al. 2003b; Behari et al. 2001; Chan et al. 1998; Semchuk et al. 1991) and decreased risk was reported in others (Tanner et al. 1989; Wang et al. 1993).

Also related to rural or farm living is the use of well water, possibly associated with runoff of pesticides or other environmental contaminants. A number of studies show a positive association (Koller et al. 1990; Morano et al. 1994; Rajput et al. 1986; Smargiassi et al. 1998; Wong et al. 1991). However, many others found no association for drinking well water and the development of PD (Behari et al. 2001; Chan et al. 1998; Engel et al. 2001; Gorell et al. 1998; Kuopio et al. 1999; Liou et al. 1997; Semchuk et al. 1991; Stern et al. 1991; Wang et al. 1993), and one found an inverse relationship (McCann et al. 1998), although many of these studies may have low power to detect outcomes, and the study designs vary.

The association between PD and exposure to metals has been intensely investigated. Welders exposed to multiple types of metals appear to be at increased risk of developing PD, particularly at an earlier age (Racette et al. 2001). One study showed a subjective association with increased frequency of heavy metal exposure; however, this could not be confirmed (Seidler et al. 1996). Other studies showed no association for occupational exposures to heavy metals in general (Gorell et al. 1999; Liou et al. 1997).

Results of epidemiologic studies examining exposure to specific metals have similarly been variable. Some studies showed a significant association with exposure to manganese alone (Engel et al. 2001; Gorell et al. 1997, 1998, 1999), and Powers et al. (2003) showed a joint effect with exposure to both manganese and iron, but others have not found any association between PD and manganese (Semchuk et al. 1993). This is supported by evidence showing no change in brain concentration of manganese (Dexter et al. 1989, 1991, 1992) in PD cases versus controls. No association was observed for exposure to iron alone (Gorell et al. 1998) but was observed for the combination of iron and copper (Gorell et al. 1997, 1998, 1999), iron and lead (Gorell et al. 1997, 1998, 1999), and iron and manganese (Powers et al. 2003). There is evidence, however, that iron (Dexter et al. 1989, 1991, 1992; Hirsch et al. 1991; Riederer et al. 1989; Sofic et al. 1988) or ferritin (Dexter et al. 1990) deposits to greater or lesser degrees in areas of the brain. Exposure to copper was found to be associated with PD (Gorell et al. 1997, 1998, 1999; Wechsler et al. 1991), although biomonitoring studies are variable, with some showing an increase (Dexter et al. 1989, 1992) and another showing no change in brain concentration (Riederer et al. 1989). Exposure to Al was found to be higher in male cases than in male controls in one study (Wechsler et al. 1991) but not in another (Semchuk et al. 1993). For mercury, one study found that a significantly larger number of PD cases had dental amalgams than did controls (Seidler et al. 1996), and an association was observed between the concentration of mercury in the blood and urine and PD (Ngim and Devathasan, 1989), but others showed no association with exposure to mercury (Gorell et al. 1998; Semchuk et al. 1993). Finally, although zinc was found in the brain tissue of patients with PD (Dexter et al. 1989, 1991, 1992), no association for zinc exposure and PD was observed in another study (Gorell et al. 1998).

Limited epidemiologic studies suggest an association between exposure to solvents and PD. One study examined exposure to organic solvents and found a statistically significant relationship to the development of PD (Smargiassi et al. 1998). In addition to the possibility of solvents causing PD, Pezzoli et al. (2000, 2004) found that exposure to hydrocarbon solvents increased PD severity and earlier age at onset; another showed suggestive evidence of an association between solvents and PD (Seidler et al. 1996).

There are claims of an increase of PD for those working with wood or in other forms of construction. One study found a nonsignificant but highly elevated occupational risk (Hertzman et al. 1990); another found a link to exposure to wood preservatives (Seidler et al. 1996); and a third found an increased risk for those having worked on construction sites (Herishanu et al. 2001). However, the agent associated with PD is not determined for this exposure.

## Parkinsonian Syndromes

There are other neurodegenerative conditions with symptoms similar to PD, including MSA and PSP. These diseases often can be confused with PD because they have similar symptoms and may also co-exist (Drayer et al. 1986). Therefore, it is important to consider whether they have similar or distinct etiologies compared with PD and other neurodegenerative diseases.

MSA is a cluster of three related disorders, one of which is parkinsonian that is characterized by low blood pressure resulting in dizzy spells. The incidence rate is about 0.6 in 100,000 per year, with the incidence rate increasing to 3 in 100,000 in the population older than 50 years (Bower et al. 1997; Wenning et al. 2004). The evidence of an association with environmental agents is limited. Nee et al. (1991) found MSA to be significantly associated with metal dusts and fumes, plastic monomers and additives, organic solvents, and pesticides when compared with controls. Case–control studies of biomarkers of exposure show increased concentrations of iron in the brains of patients with MSA (Dexter et al. 1991, 1992) and in patients diagnosed with both PD and MSA (Drayer et al. 1986) versus controls. No change in brain concentration of manganese was observed (Dexter et al. 1991, 1992).

PSP is a neurologic condition affecting the brainstem that also has symptoms similar to those of PD. It is characterized by movement and visual abnormalities. Little is known about the incidence rate (Bower et al. 1997) or etiology of PSP (Davis et al. 1988; Golbe et al. 1996; Pezzoli et al. 2004; Rajput and Rajput 2001; Rehman 2000). Case–control studies examining the body burden of exposure shows iron was increased in the brains of patients with PD (Dexter et al. 1991, 1992) and patients with both PD and PSP (Drayer et al. 1986). However, neither iron nor Al in the brains of cases was found to be different from cases in another study (Hirsch et al. 1991). Copper was decreased in the brains of patients with PD (Dexter et al. 1991, 1992) and manganese had no effect (Dexter et al. 1991, 1992).

## Amyotrophic Lateral Sclerosis

Amyotrophic lateral sclerosis (ALS), also known as motor neuron disease or Lou Gehrig’s disease, is a rare neuromuscular disease with an incidence rate of about 1 in 100,000. It is characterized by muscular weakness from the degeneration of motor neurons, and like PD, intellect and personality is often unaffected. The National Institute of Neurological Disorders and Stroke reports that only 5–10% of all ALS cases can be traced to genetics, particularly to a mutation related to the superoxide dismutase 1 enzyme. This leaves the vast majority of cases without a known etiology, with the potential for environmental association briefly outlined below.

Far fewer studies have examined the association of pesticides and ALS than for both AD and PD. McGuire et al. (1997) found that agricultural chemicals have a significant association with the development in ALS, with a stronger association for men than for women.

Metals may play a role in the development of ALS. Some studies have observed an association with occupation in welding or soldering (Armon et al. 1991; Gunnarsson et al. 1992), but not all have found metals to be related to ALS (Gresham et al. 1986; McGuire et al. 1997). More specifically, an association has been observed with exposure to lead (Armon et al. 1991; Chancellor et al. 1993; Felmus et al. 1976; Kamel et al. 2002), but no association was observed between ALS and lead levels in various tissues (Kapaki et al. 1989; Stober et al. 1983) or toenails (Bergomi et al. 2002); however, these studies had limited numbers of study participants. No association was observed between exposure to zinc and ALS (Vinceti et al. 2002), and the evidence from biomarker studies is inconclusive, with an increased (Gellein et al. 2003), decreased (Yasui et al. 1993), and no association observed for levels in brain tissue (Kapaki et al. 1997; Nagata et al. 1985) or toenails (Bergomi et al. 2002) compared with controls. However, these studies may have had limited power based on the size of the study population. Although one epidemiologic study showed no association between exposure to copper and ALS (Vinceti et al. 2002), there was decreased copper concentration observed in both cerebrospinal fluid and blood (Kapaki et al. 1997), and no association in toenails (Bergomi et al. 2002) among patients with ALS versus controls. Mercury was associated with ALS risk (Felmus et al. 1976) but was found in lower concentrations in the blood of ALS patients versus controls (Moriwaka et al. 1993).

Case–control studies examining biomarkers of iron, manganese, selenium, and Al and risk of ALS were found. Increased iron levels have been observed in brain tissue (Kasarskis et al. 1995; Yasui et al. 1993), although not in blood (Nagata et al. 1985) or toenails (Bergomi et al. 2002). An increase of manganese was observed in cervical cords (Miyata et al. 1983), both an increase (Kapaki et al. 1997) and decrease (Nagata et al. 1985) in blood levels, and no difference in toenail concentration (Bergomi et al. 2002) among cases versus controls. Selenium was found to be increased (Nagata et al. 1985) and decreased (Moriwaka et al. 1993) in blood cells, but no association was observed in toenails (Bergomi et al. 2002) of patients with ALS versus controls. An increase was observed in Al in central nervous system tissue (Yasui et al. 1991a, 1991b) and cerebrospinal fluid (Sood et al. 1990), yet others observed no association in spinal cords (Kasarskis et al. 1995) or toenails (Bergomi et al. 2002). However, the latter two studies had small numbers of study participants, possibly limiting the power to detect an association.

A few studies found a relationship between other exposures and ALS. Gunnarsson et al. (1992) found a nonsignificant association with solvents, but the association was stronger and statistically significant for males with family history of neurodegenerative disease or thyroid disease. Others found conflicting results (Chancellor et al. 1993; McGuire et al. 1997). One study found that those with a history of occupation in the manufacturing of plastics have a significant association with the development of ALS (Deapen and Henderson et al. 1986). Occupations in electrical work have been implicated in the development of ALS in a few studies (Deapen and Henderson 1986; Gunnarsson et al. 1992).

## Conclusion

Epidemiologic evidence for an association between environmental agents and neurodegenerative disease is inconclusive. The amounts of xenobiotics released into the environment are huge by any measure, and the paucity of information about their effects on various physiologic systems, including neurodevelopmental processes, represents a major gap in knowledge. To close this gap, the following broad areas of research topics need attention: *a*) better health tracking and monitoring data for chronic diseases, *b*) more comprehensive and longitudinal biomonitoring of environmental agents that can be linked with specific molecular/biochemical markers of exposure and subsequent health outcome data, and *c*) more epidemiologic research and testing of environmental agents to better define their effects on the adult and developing brain, as well as other critical organ systems.

Until such time that ethically and scientifically well-designed epidemiologic studies can provide a reasonable certainty that specific environmental agents, either alone or in combination with other agents, cause a given neurodegenerative disease, research on the environmental contribution to neurodegenerative disease needs to continue.
